# Variations on Negative Stain Electron Microscopy Methods: Tools for Tackling Challenging Systems

**DOI:** 10.3791/57199

**Published:** 2018-02-06

**Authors:** Charlotte A. Scarff, Martin J. G. Fuller, Rebecca F. Thompson, Matthew G. Iadaza

**Affiliations:** ^1^Astbury Centre for Structural Molecular Biology, University of Leeds; ^2^Astbury Biostructure Laboratory, University of Leeds

**Keywords:** Biochemistry, Issue 132, Electron microscopy, Negative stain, Biological TEM, Blotting, Electron microscopy grids, Carbon coating

## Abstract

Negative stain electron microscopy (EM) allows relatively simple and quick observation of macromolecules and macromolecular complexes through the use of contrast enhancing stain reagent. Although limited in resolution to a maximum of ~18 - 20 Å, negative stain EM is useful for a variety of biological problems and also provides a rapid means of assessing samples for cryo-electron microscopy (cryo-EM). The negative stain workflow is straightforward method; the sample is adsorbed onto a substrate, then a stain is applied, blotted, and dried to produce a thin layer of electron dense stain in which the particles are embedded. Individual samples can, however, behave in markedly different ways under varying staining conditions. This has led to the development of a large variety of substrate preparation techniques, negative staining reagents, and grid washing and blotting techniques. Determining the most appropriate technique for each individual sample must be done on a case-by-case basis and a microscopist must have access to a variety of different techniques to achieve the highest-quality negative stain results. Detailed protocols for two different substrate preparation methods and three different blotting techniques are provided, and an example of a sample that shows markedly different results depending on the method used is shown. In addition, the preparation of some common negative staining reagents, and two novel Lanthanide-based stains, is described with discussion regarding the use of each.

**Figure:**
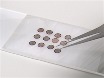


## Introduction

Despite recent attention to the resolution revolution resulting from significant advances in cryo-electron microscopy^1^ (cryo-EM), negative stain EM remains a powerful technique and a crucial component of electron microscopists' toolbox. Negative staining still remains the best method for rapid assessment of a sample before optimizing cryo-grid conditions^2^. The high contrast and speed of grid preparation of negative stained samples makes it ideal for assessing sample purity, concentration, heterogeneity, and conformational flexibility^3^. Many biologically informative structures have resulted from negative stain reconstructions, despite the technique's resolution being limited to ~18 Å resolution^4^,^5^,^6^, and some samples yield better results in stain than cryo-EM for a variety of reasons^7^.

In negative stain EM, the particle of interest is adsorbed onto the surface of an EM grid and enveloped by an amorphous matrix of electron dense stain compound. A high relative contrast is produced between the background and the particle of interest, with the particle being less electron dense than the surrounding stain^8^. The particles appear as light areas because of their low electron scattering power relative to the dense surrounding stain, which scatters the electrons more and appears darker. Substructural features of particles can be deduced from the detailed examination of resultant images as stain will penetrate into any crevice and produce irregular contrast detail^9^.

The negative staining process begins with the preparation of a support substrate on which the sample particles are captured, and the layer of dried stain supported. The most commonly used support substrate is a layer of amorphous carbon, sometimes supported by a thin layer of polyvinyl (*e.g.* Formvar) or nitrocellulose (*e.g.* Collodion) polymer. These substrates can be purchased commercially or prepared in-house using the protocols described below.

After the support substrate is prepared, the sample can be applied, and the excess solution blotted off. Samples should be suspended in a suitable buffer for negative-staining. It is best to avoid the use of phosphate buffer and high salt concentrations, which can give rise to crystalline precipitates that can obscure the specimen. Reducing agents, detergents, sucrose, glycerol, and high concentrations of nucleotide should also be avoided as they also affect stain quality^4^. When the buffer composition cannot be changed, washing the surface of the EM grid with water or a more suitable buffer after adsorption and prior to staining may reduce the formation of buffer related artifacts and generally improve the stain background. If buffer artifacts are suspected, it can be informative to stain a buffer-only grid to determine if the buffer components are the source of the observed artifacts.

After the sample is adsorbed, and blotted and washed if necessary, a staining reagent is applied. A variety of reagents have been found to be effective negative stains (**Table 1**), but the stain must be chosen to suit the sample. A 'halo' of stain forms around the particle due to both the displacement of the stain molecules by the hydrophobic regions of the protein and repulsion by charged groups. Therefore, the stain must be chosen so that the protonation state of any potential charged groups on the protein is the same as the stain at the working pH. Opposite charges on the surface of the protein can contribute to a positive staining effect, which although a useful technique in its own right^10^ is not in the scope of this paper. The most commonly used negative staining reagents are uranyl acetate and uranyl formate. These stains have a relatively fine grain size (4 - 5 Å)^9^ and provide higher resolution images over other stains such as phospho-tungstates (8 - 9 Å grain size)^9^,^11^, ammonium molybdate^11^, and some lanthanide-based stains^12^. Uranyl acetate and formate also act as a fixative, preserving many protein-protein interactions on a millisecond time scale^13^, although the low pH of the stain and its propensity to precipitate at physiological pH may be detrimental to some samples^14^. Despite their utility, the uranyl salts also present logistical challenges as they are both toxic and mildly radioactive, which can require special handling, storage, and disposal requirements, which leads some users to seek non-radioactive alternatives.

There are a large variety of methods described for substrate preparation, sample application, and staining of EM grids. The most appropriate method to use is sample dependent and can be difficult to ascertain when tackling a new system. This manuscript describes two methods of substrate preparation and three blotting methods; side blotting, flicking^5^,and rapid flushing^15^. Side-blotting is the simplest of the methods described. Both the flicking method and the rapid flushing method are more difficult to implement but limit the contact time of the sample with the support film before fixation and have been shown to ameliorate formation of stain artifacts for some samples^5^. The goal of this manuscript is thus to provide an initial workflow for tackling the visualization of challenging systems by negative-stain EM.

## Protocol

### 1. Preparation of EM Grids

1. **Carbon Sheet Method**
  1. Prepare a freshly cleaved mica sheet.
    1. Gently insert a precision syringe needle or a razor blade at one corner of the mica sheet, a few mm in between the layers. Insert the tool as close to the vertical centre of the sheet as possible to produce two pieces of approximately equal thickness.
    2. Carefully prise apart the two halves of the mica sheet. Do this can by eye, or under a dissecting microscope.
    3. Cut off one of the corners of each of the newly cleaved mica sheets. In the event that the sheet turns over in the carbon evaporator during vacuum release, the carbon coated side of the sheet can be identified.
  2. Place the cleaved mica sheet/s in the chamber of a carbon evaporator, with the freshly-cleaved surface facing up.
  3. Make sure the carbon evaporator is set-up correctly with a properly prepared carbon electrode. NOTE: The method of preparing the carbon rods will vary depending on the specifications of the carbon evaporator. A protocol for one instrument is as follows until step 1.1.5.
    1. Sharpen a carbon rod with a sharpener to have a sharp tip and then polish it with a paper towel to remove any rough burrs.
    2. Using fine sandpaper flatten the end of a second rod and again polish it smooth with a paper towel.
    3. Place the two carbon rods into the evaporator according to manufacturer's instructions. Ensure the sharpened end on the first rod makes firm contact with the flattened face of the second rod.
  4. Place a small piece of clean, dry filter paper partially under the mica if the carbon thickness is going to be gauged visually. Alternatively, place a white frosted microscope slide with a small dab of vacuum grease alongside the mica to gauge carbon thickness.
  5. Deposit carbon onto the mica according to manufacturer's instructions.
    1. Pump the vacuum down and wait until it is at 10^-5^ mbar. Set the voltage of the electrode to 4.0 V (up to 5 V may be required depending on the carbon rod source).
    2. Run multiple short pulses of approximately 3.5 s in duration through the electrode to deposit 1-2 nm thick evaporations of carbon on the mica surface. NOTE: As current is applied to the carbon rod it will glow red and then white. Do not stare at the bright light as this could damage your eyes.
    3. Allow the carbon to deposit on the mica until the desired thickness is reached, as measured by the carbon evaporator's thickness gauge or by visual observation of the carbon deposited on the filter paper or microscope slide. Ensure that the final carbon layer is 5-10 nm thick.
      1. If the carbon thickness is gauged visually compare the frosted part of the microscope slide coated with vacuum grease to the exposed area, it will become darker as more carbon is deposited. NOTE: There is no quantitative method to determine carbon thickness when using this method.
  6. Vent the vacuum chamber and remove the carbon-coated mica from the carbon evaporator. NOTE: The carbon-coated mica can be left to settle overnight before proceeding with subsequent steps
  7. Use one of the two water containers to float the carbon film onto the EM grids: a container with a drain valve at the bottom so water can be drained out and the carbon layer lowered on to the awaiting grids or a lifting rig that the grids can be set on under the water surface, which can subsequently raise the grids up to the carbon film at the water's surface.
  8. Fill the container with ultrapure distilled water so that the surface of the water is approximately 5 mm from the top. Clean the surface of water by dragging a sheet or two of lens tissue over the surface to remove any floating particulates.
  9. Place a piece of clean stainless-steel mesh (1 inch by 2.5 inches is an appropriate size) under the surface of the water.
  10. Using a pair of fine tweezers, lay clean, dry EM grids face up (according to the manufacturer's description) on the stainless-steel mesh. Pack the grids together as tightly as possible, but do not allow them to overlap.
  11. Once the grids are arranged, firmly grip the carbon coated mica sheet in a pair of tweezers or film developing tongs.
  12. Introduce the mica sheet into the water. Ensure that this is done at a very shallow angle (~10 degrees). NOTE: The mica should break through the water surface and submerge, whilst the carbon film should separate from the mica and float on the water surface. This step should not be performed directly over the grids, to avoid damage and/or contamination.
    1. To minimize the possibility of the carbon film will not separating from the mica sheet, score around the edge of the mica sheet with a razor blade or cut one corner off with small scissors before introducing it into the water.
  13. Once the carbon film has detached, remove the mica sheet or let it fall to the bottom of the container.
  14. Using fine tipped tweezers, apply very gentle pressure and with slow movements guide the carbon film over the top of the grids.
  15. Bring the carbon sheet in contact with the surface of the grids either by slowly draining the water or raising the lifting ring, depending on the type of apparatus used.
  16. Carefully lift the stainless-steel mesh (now with carbon-coated grids) from the apparatus and wick away some of the excess water using a piece of filter paper. Ensure that this is done by touching the filter paper to the very edge of the steel mesh but not coming in contact with the grids or carbon film.
  17. Place the mesh of grids in a petri dish containing a dry piece of filter paper and allow to it dry completely. NOTE: This is best affected by drying overnight at room temperature, but the step can be expedited by placing the grids in an oven at approximately 60 °C.
2. **Float and coat (direct carbon deposition). This method has been described in detail previously** 16
  1. Completely fill a clean large glass bowl to the brim with distilled water so a meniscus forms at the top.
  2. Apply a single drop of collodion solution (nitrocellulose in amyl acetate) to the surface of the water using a clean pasteur pipette, allow the droplet to spread out and dry completely. Once dry a thin layer of collodion floating on top of the water surface will be visible.
  3. Gently remove the collodion layer using a toothpick to remove dust or other contamination from the surface of the water.
  4. Apply a second collodion droplet to the water and allow it to spread out and dry for 2-3 minutes. NOTE: Repeat steps 1.2.3-1.2.4 until a flat and wrinkle free sheet of collodion is obtained.
  5. Using a pair of fine tweezers place EM grids face down (according to the manufacturer's description) on the floating collodion sheet. Pack the grids together tightly in a hexagonal array, but do not allow them to overlap. NOTE: If a grid is misplaced or placed upside down it is generally best to leave it in place rather than risk damaging the collodion sheet when trying to move it.
  6. Once all of the grids are placed, gently lay a sheet of filter paper over them. Allow the paper to become saturated by capillary action. NOTE: Any size or thickness of filter paper is appropriate if it completely covers the grids.
  7. Use a toothpick to remove any collodion film that extends beyond the filter paper.
  8. Grip the filter paper at the edge and peel it from the water surface. NOTE: The grids should stay adhered to the paper.
  9. Place the paper flat and collodion-face up in a petri dish and allow it to dry completely.
  10. Place the filter paper with the grids in the chamber of a carbon evaporator with a properly prepared carbon electrode as detailed in 1.1.2.
  11. Follow the carbon evaporation procedure as described for the carbon sheet method in
3. Allow several seconds between pulses to avoid overheating and damaging the nitrocellulose sheet. NOTE: If desired the polymer layer can be removed after the grids have been carbon coated, although this step is rarely necessary. Place the grids carbon side up on a fresh piece of filter paper and put several drops of acetone on the paper near, but not on, the grids. Allow the acetone to spread out under the grids and dissolve and absorb the polymer layer.

### 2. Preparation of Negative Staining Reagents

1. **Preparation of Uranyl Acetate**
  1. Bring a small volume of ultrapure water to a boil and allow it to boil for 10 min to thoroughly degas. Allow it to cool slightly, and then use it to dissolve uranyl acetate (UA) at 1-2 % (w/v). NOTE: Perform this procedure in a fume cupboard and with appropriate personal protective equipment.
  2. After the solution has cooled, filter through a 0.2 µm syringe filter or filter paper.
  3. Store the UA protected from light and at 4 ˚C. The solution is stable for up to 1 year.
2. **Preparation of Uranyl Formate from Powder. This Method has been Described in Detail Previously** 8
  1. Dissolve 20 mg uranyl formate (UF) powder in 2 mL of boiled degassed ultrapure water (as in step 2.1.1) by stirring.
  2. While continuing to stir, add 8 µL of 5 M NaOH, the solution should change to a darker yellow colour, but no precipitate should form.
  3. Filter the solution through a 0.2 µm syringe filter.
  4. Store the UF stain protected from light. Discard the stain should if any precipitate or brown discoloration is observed. The solution is only stable for 1-2 days.
3. **Preparation of Uranyl Formate from Uranyl Acetate**
  1. Precipitate 1 mL of 1% (w/v) UA stain by adding 100 µL of 1 M NaOH.
  2. Centrifuge the mixture for 2 min at maximum speed in a benchtop centrifuge.
  3. Discard any supernatant and dissolve the precipitate in 100 µL of 5% (v/v) formic acid by vigorous vortexing.
  4. Dilute to a final volume of 1 mL with 900 µL ultrapure water to yield the UF stain in 0.5% (v/v) formic acid.
  5. Store the UF stain protected from light. Discard the stain if any precipitate or brown discoloration is observed.
4. **Preparation of Other Staining Reagents**
  1. **Preparation of Lanthanide Acetate Stains**
    1. Dissolve Samarium Acetate (SmAc), Gadolinium Acetate (GdAc), Thulium Acetate (TmAc), or Erbium Acetate (ErAc) at 1-2% (w/v) in ultrapure water. NOTE: If samples show positive staining or poor adherence to the grid when using these stains, they can be acidified with up to 0.5% (v/v) formic acid. Positive staining results in the sample appearing as a dark object surrounded by a white halo. Poor adherence to the grid will result in fewer molecules than expected being observed on the grid.
  2. **Preparation of Ammonium Molybdate and Sodium Phosphotungstate**
    1. Dissolve the stain at 1-3% (w/v) in ultrapure water. Adjust the pH to 7.0 using 5 M NaOH if desired.

### 3. Adsorbing Samples to the Carbon Substrate and Staining

1. **Preparation of the Grid Surface for Sample Application by Rendering it Hydrophilic**
  1. Place the grid facing up on a microscope slide in a glow discharge unit.
  2. Treat the grid for a minimum of 30 s at 10 mA. NOTE: The exact method of glow discharge will depend on the specifications of the particular piece of equipment used.
  3. Alternatively this may be accomplished by UV irradiation for 10 minutes using a benchtop UV lamp^4^.
2. **Side Blotting Method. This Method has been Described in Detail Previously** 8
  1. Grip the edge of the grid with a pair of negative pressure tweezers, and apply 3-5 µL of sample to the support surface.
  2. Allow the sample to adsorb to the grid surface for 10 s to 1 min. Optimize the adsorption time must for individual samples.
  3. Touch the edge of the grid to a sheet of filter paper and allow capillary action to pull off the liquid.
  4. Optional: Wash the grid. Place 50 µL drops of ultrapure water or appropriate volatile buffer solution on a sheet of laboratory film. Gently touch the carbon surface of the grid to the drop and lift off a small droplet onto the surface of the grid. Touch the edge of the grid to a sheet of filter paper and allow capillary action to pull off the liquid.
  5. Repeat this wash step as many times as desired.
  6. Place two 50 µL drops of staining reagent on a sheet of laboratory film.
  7. Gently touch the carbon surface of the grid to the drop and lift off a small droplet onto the top surface of the grid. NOTE: If the stain migrates to the back of the grid then the grid should be discarded.
  8. Touch the edge of the grid to a sheet of filter paper and allow capillary action to draw off the liquid. Perform this staining step twice.
  9. Allow the grid to air dry or dry under an incandescent lamp.
3. **Flicking Method**
  1. Grip the edge of the grid with a pair of negative pressure tweezers, and apply 3-5 µL of sample to the support surface.
  2. Holding the tweezers in one hand, so that the grid is angled at approximately 45° facing away, rapidly flick the wrist of that hand to 'flick off' the majority of the droplet that is on top of the grid.
  3. Optional: Using a glass Pasteur pipette apply a drop of wash solution to the support surface and 'flick off' as in 3.2.2. Repeat as necessary.
  4. Using a glass Pasteur pipette apply a drop of staining reagent to the support surface and 'flick off' as in 3.2.2. Repeat 1-3 times dependent on stain depth required for visualization of specimen. NOTE: This is not the only factor that attributes to final stain depth (see discussion).
  5. Remove excess stain by touching the torn edge of a piece of filter paper to the edge of the grid.
  6. Allow the grid to air dry or dry under an incandescent lamp.
4. **Rapid Flushing Method**
  1. Draw 30-70 µL of stain (1 % UA usually used) into the tip of a 200 µL pipette, turn the volume dial to draw up 5 µL of air, then draw up wash/mixing reagent (5-30 µL), if required, followed by another small air gap and then draw up 5 µL of sample.
  2. Grip the edge of a grid with a pair of negative pressure tweezers, holding the tweezers so that the grid is angled at approximately 45° facing away from the researcher, eject the entire contents of the pipette tip across the face of the carbon-coated EM grid.
  3. Remove excess stain by touching the torn edge of a piece of filter paper to the edge of the grid.
  4. Allow the grid to air dry or dry under an incandescent lamp. NOTE: For all methods it is advisable to slide the torn edge of a sheet of filter paper along the forceps until it reaches the grid as this removes solution trapped between the two sides of the forceps, which can pull the dried grid into the jaws of the forceps once they are opened. The grid in the tweezers can also be placed on the edge of a fume hood to dry. The constant airflow can help produce more even staining.

## Representative Results

All of the staining reagents tested produced negative staining to some degree, with UF yielding the samples with the greatest contrast and sharpest, most detailed particles. For deeply embedded samples (Figure 1) lanthanide based stains ErAc and TmAc produced negative staining of equivalent quality to UA as judged by the apparent contrast and sharpness of the stained particles, with TmAc producing clearer, more crisp images than ErAc. Although the larger grain size of TmAc becomes apparent at high magnification, when Tobacco Mosaic Virus (TMV) particles were stained with 1% TmAc the ~23 Å repeat of the TMV particle^17^ was still clearly visible by eye and as a meridional layer line in the Fourier transform of the raw image. None of the other lanthanide stains tested, ErAc, SmAc, or GdAc, were able to resolve this feature. Class averages were generated by extracting overlapping segments from TMV particles where the helical repeat was visible. The extracted segments were then aligned and classified using RELION^18^ to better visualize the periodic feature (Figure 2).

Some samples are especially sensitive to the method of staining, such as the muscle derived C-protein. C-protein, which consists of a flexible string of Ig and Fn-like domains, produces significantly different images by negative-stain EM dependent on the method of staining used (Figure 3). When using the side-blotting method, collapsed ring-like structures are observed, whereas when stained by the rapid flushing or flicking methods, C-protein is observed as a series of domains that resemble beads on a string.

**Table:** 

**Reagent**	**Concentration**	**pH **	**Type**
Ammonium Molybdate	1 - 2 %	5 – 7	Anionic
Erbium acetate (ErAc)	1 – 2%	6	Cationic
Gadolinium Acetate (GdAc)	1 – 2%	6	Cationic
Methylamine tungstate	2%	6 – 7	Anionic
Samarium acetate (SmAC)	1%	6	Cationic
Sodium silicotungstate	1 – 5 %	5 – 8	Anionic
Sodium phosphotungstate	1 -3 %	5 – 8	Anionic
Thulium Acetate (TmAc)	1 – 2%	6	Cationic
Uranyl Acetate (UA)	1 – 3%	3 – 4	Cationic
Uranyl Formate (UF)	0.75 – 1%	3 – 4	Cationic

**Table 1: Some common negative staining reagents.**

**Figure:**
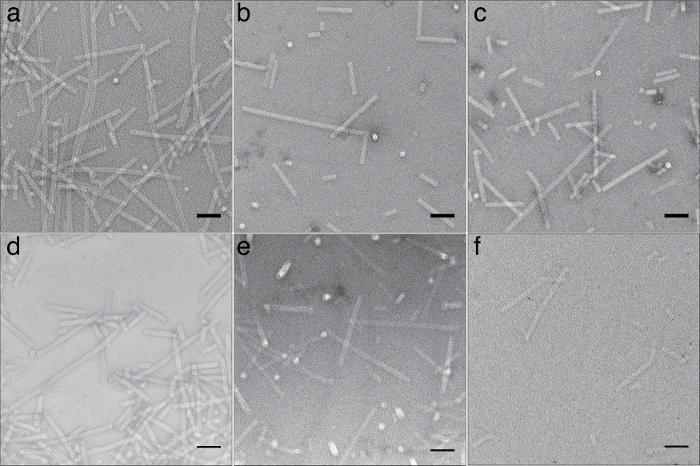

**Figure 1: Example micrographs of Tobacco Mosaic Virus stained with various negative stain reagents(A)** 1% UF **(B)** 2.5% TmAc **(C)** 2.5% ErAc. **(D)** 1% UA **(E)** 2.5% GdAc and **(F)** 2.5% SmAc. Scale bars are 100 nm. Representative images from multiple replicates with multiple areas imaged per replicate. Please click here to view a larger version of this figure.

**Figure:**
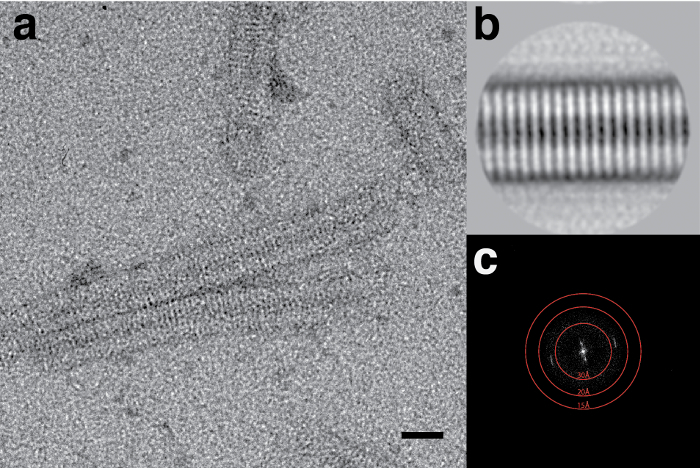

**Figure 2: Staining Tobacco Mosaic Virus with Thulium acetate(A)** High magnification of area from a micrograph of TMV stained with 1% TmAc. Scale bar is 20 nm. **(B)** Class average of extracted TMV segments. **(C)** Fourier transform of the image in panel A showing layer line reflections at ~23 Å. Please click here to view a larger version of this figure.

**Figure:**
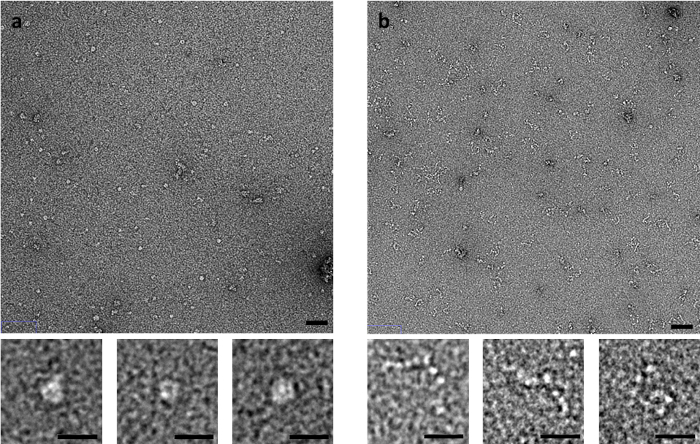

**Figure 3: Effects of blotting method on the conformation of C-protein.(A)** C-protein stained with UA using the side blot method and **(B)** flicking method. Upper panel scale bar is 50 nm, lower panel scale bar is 20 nm. Representative images from multiple replicates with multiple areas imaged per replicate. Please click here to view a larger version of this figure.

## Discussion

This manuscript describes multiple methods for negative staining of samples for electron microscopy using a variety of staining reagents, including two novel lanthanide reagents (TmAc and ErAc). Many of the steps of the negative staining process must be optimized for individual samples including the choice of stain, amount of washing required if any, and the blotting technique. This manuscript thus provides a basis for microscopists to develop their own workflows for tackling the negative-staining of challenging systems.

The choice of stain is highly sample dependent. Samples that are especially sensitive to low pH may be degraded by UA and/or UF, despite the fixative properties of these stains^19^. In these cases, lanthanide based stains such as TmAc or ErAc may be more appropriate, although the overall pH of the preparation must be kept below the isoelectric point of the sample protein to help prevent positive staining. This can be accomplished by acidifying the stain with acetic acid if necessary. For especially low pH sensitive samples, anionic tungstate or molybdate stains may be more effective. Although these stains have been found to induce the formation of artifacts in some cases, such as the formation of rouleaux in lipoprotein samples^20^. Again, the pH of the stain may need to be adjusted, this time to above the isoelectric point of the sample, to prevent positive staining.

Washing of the sample prior to staining may be necessary if the buffer in which the specimen is maintained has a high salt or phosphate component. In many cases, washing can be performed with ultrapure water but for more sensitive samples, which may degrade or undergo structural changes when exposed to water alone, washing may need to be performed with a volatile buffer of low ionic strength^8^. Even under carefully controlled conditions, washing can result in some structural rearrangement on the carbon surface^21^.

The method by which a grid is prepared in terms of sample adsorption, blotting and staining can also significantly affect what is observed. The most appropriate method is thus, again, highly sample dependent. C-protein, for example, is observed as a globular ring-like structure following side-blot staining, but this appears to be an artifact of the staining process, as revealed when grids are prepared by the flicking method (or by the rapid flushing method) (Figure 3). In the flicking and rapid flushing methods, the time the sample has to interact with the carbon support surface before fixation is minimized^15^. The sample also experiences fewer forces from the receding meniscus upon blotting before fixation. This means that structural changes in the specimen that could occur upon prolonged absorption time on the carbon film or through capillary action are minimized. The rapid flushing method can also be used for time-resolved analysis of specimens. The sample can be mixed with a ligand or additive within a pipette tip for a set period of time before application to a grid or only momentarily on the grid surface before fixation within milliseconds.

The depth of stain required to provide optimal images of a particular specimen is again sample dependent^2^. If the stain is too shallow, molecules can be damaged by the electron beam but if the stain is too thick structural features can be lost. Stain depth is influenced by multiple factors such as hydrophilicity of the grid surface, evenness of the carbon layer, the amount of stain applied to the grid, the length of time stain is in contact with the grid prior to blotting, the extent of blotting and the time it takes for the grid to completely dry. A grid will never have an even distribution of stain across its entirety and therefore areas of the grid appropriate for imaging need to be selected carefully. Indeed, grids often vary in quality even when prepared on the same day under the same conditions. A good example of how variation in stain depth affects the appearance of molecules and the appropriate stain depth for imaging is provided by Burgess *et al^5^.*

Despite negative staining being a very versatile, quick, and simple method, not all biological specimens are amenable to visualization by this method. Fragile assemblies can collapse or disassemble upon adsorption, staining or drying on the EM grid^22^. Negative staining can also lead to flattening of molecules and induce preferred orientations of molecules on the carbon support film^7^.

Negative stain is a valuable tool for assessment of specimens in its own right and also prior to cryo-EM analysis but many of the physical forces the sample encounters during the process are poorly understood. Therefore, the best approach to use is highly sample dependent and must be determined by trial-and-error rather than taught following a fixed protocol.

## Disclosures

The authors declare no competing financial interests.
